# Predictors of Metastasis in 68GA-Prostate Specific Membrane Antigen Pet-CT in the Primary Staging of Prostate Cancer

**DOI:** 10.3390/jcm13102774

**Published:** 2024-05-08

**Authors:** Erkin Karaca, Erdem Kisa, Mehmet Caglar Cakici, Taha Cetin, Mehmet Yigit Yalcin, Mert Hamza Ozbilen, Cagdas Bildirici, Gokhan Koc

**Affiliations:** 1 Department of Urology, Izmir City Hospital, Izmir 35540, Turkey; 2 Department of Urology, Izmir Medicana International Hospital, Izmir 35170, Turkey; drerdemkisa@hotmail.com; 3 Department of Urology, Istanbul Medeniyet University, Istanbul 34720, Turkey; mcaglarcakici@hotmail.com; 4 Department of Urology, Izmir Medicalpoint Hospital, Izmir 35575, Turkey; tahacetin88@gmail.com (T.C.); gokfekoc@gmail.com (G.K.); 5 Department of Urology, Sakarya Sadika Sabanci Hospital, Sakarya 54580, Turkey; yigityalcin@hotmail.com; 6 Department of Urology, Adana City Hospital, Adana 1370, Turkey; merthozbilen@hotmail.com; 7 Department of Urology, Bitlis State Hospital, Bitlis 13000, Turkey; cagdas_bildirici@hotmail.com

**Keywords:** prostate, prostate cancer, PSMA, prostate specific membrane antigen, PET-CT

## Abstract

**Background**: The objective of this study was to investigate factors influencing Gallium 68 Prostate Specific Membrane Antigen Positron Emission Tomography (Ga68 PSMA PET-CT) uptake for primary staging in prostate cancer. **Methods**: Retrospective analysis was conducted on 499 non-metastatic and 243 de novo metastatic prostate cancer cases undergoing Ga68 PSMA PET-CT. Demographic, clinical, and imaging data were collected and analyzed. Multivariate logistic regression determined independent risk factors for metastasis detection on Ga68 PSMA PET-CT. **Results**: Metastatic cases showed higher levels of total PSA, PSA density (dPSA) and biopsy ISUP grade group compared to non-metastatic cases. Multivariate analysis identified cT2 stage and dPSA as independent predictors of metastasis detection on Ga68 PSMA PET-CT. **Conclusions**: Ga68 PSMA PET-CT plays a crucial role in prostate cancer staging, with identified factors such as clinical T stage and dPSA significantly impacting its diagnostic accuracy. These findings underscore the importance of Ga68 PSMA PET-CT in refining clinical staging and guiding treatment decisions for prostate cancer patients.

## 1. Introduction

Prostate cancer is a formidable adversary, ranked as the second most commonly diagnosed cancer worldwide and claiming the sixth spot among causes of cancer-related mortality in men [1]. The assessment of this disease typically relies on a combination of methods, including Prostate Specific Antigen (PSA) levels; digital rectal examination; and staging through the Tumor, Lymph Node, and Metastasis (TNM) classification system, alongside the European Association of Urology (EAU) risk group categorization. These tools help determine the likelihood of biochemical recurrence (BCR) following localized treatment [2,3,4]. While these traditional methods persist, the emergence of novel technologies, such as Multiparametric Prostate Magnetic Resonance Imaging (MpMRI), has revolutionized diagnostic precision and mitigated the risks associated with overdiagnosis and overtreatment [5].

The integration of MpMRI with parameters like PSA density (dPSA) has shown remarkable promise in reducing unnecessary biopsies and enhancing local staging accuracy, marking a substantial advancement in prostate cancer management [6,7]. There are other tools to detect clinically significant prostate cancer, the like prostate health index (PHI), which is a formula that combines total PSA, free PSA and -2proPSA [8]. Combining PHI with MpMRI has also been shown to help estimation of the risk category of prostate cancer at initial diagnosis [9].

As the landscape of prostate cancer diagnosis and management continues to evolve, there is a burgeoning interest in optimizing imaging modalities for precise disease characterization. Among these modalities, Gallium 68 Prostate Specific Membrane Antigen Positron Emission Tomography (Ga68 PSMA PET-CT) has captured considerable attention for its potential in detecting systemic metastasis [10,11,12]. Ga68 PSMA PET-CT has the capacity to revolutionize the diagnostic paradigm by providing clinicians with unprecedented insights into disease extent and treatment response, potentially reshaping therapeutic decision making in prostate cancer management.

Recent studies have underscored the robust prognostic utility of PSMA PET-CT in managing patients in both the primary setting and after BCR, particularly in cases exhibiting a strong PSA response [13]. Moreover, Ga68 PSMA PET-CT has emerged as a predictive imaging modality for patients with advanced prostate cancer who have undergone Lu-PSMA-617 radionuclide therapy, demonstrating its versatility across various stages of the disease [14]. Notably, a prospective observational study highlighted the critical role of PSMA PET-CT in patients undergoing radical treatment without prior biopsy, emphasizing its significance in treatment planning and monitoring [15].

Motivated by the imperative to enhance diagnostic accuracy and streamline treatment strategies, this study seeks to delve into the multifaceted factors influencing Ga68 PSMA PET-CT uptake for primary staging in prostate cancer cases. Through a comprehensive investigation into these determinants and meticulous analysis of imaging outcomes, we aspire to contribute to the ongoing refinement of imaging protocols. By fostering a deeper understanding of Ga68 PSMA PET-CT’s pivotal role in optimizing clinical outcomes for patients with prostate cancer, we aim to pave the way for more personalized and effective approaches to diagnosis and management. Ultimately, our efforts are directed towards advancing the field of prostate cancer care, with the overarching goal of improving patient outcomes and quality of life.

## 2. Methods

### 2.1. Study Design

Our study, conducted with the explicit approval of the ethics committee at the University of Health Sciences Izmir Tepecik Training and Research Hospital (Decision No: 2021/10-38), was meticulously designed to investigate the efficacy of Ga68 PSMA PET-CT scans in prostate cancer diagnoses. The study spanned from October 2020 to October 2021 and included patients who underwent imaging at the Nuclear Medicine unit of our hospital. Rigorous exclusion criteria were applied to ensure the homogeneity and reliability of the study. Cases undergoing imaging for restaging purposes were excluded, as were those with missing data or alternative diagnoses, such as Small Cell Prostate Carcinoma or High-Grade Prostatic Intraepithelial Neoplasia. Moreover, only cases with confirmed Prostate Acinar Adenocarcinoma pathology from transrectal ultrasound-guided prostate biopsy (TRUS-BX) were included. Cases whose tissue diagnosis was obtained by Transurethral Resection of Prostate (TURP), Simple Prostatectomy, or biopsy of metastasis were excluded. The final cohort comprised 742 prostate cancer cases, divided into 499 without metastasis at primary staging and 243 presenting with de novo metastatic disease.

### 2.2. Data Collection

Comprehensive data collection was conducted for all patients enrolled in the study. Each patient underwent a meticulous anamnesis and physical examination, which included a thorough digital rectal examination. Prostate biopsies were performed under transrectal ultrasound guidance to confirm the presence and characterize the nature of the cancerous lesions. In cases categorized as low risk according to the EAU risk classification, PSMA-PET CT scans were selectively requested based on patient-reported symptoms, such as bone pain, or if suspicious areas were identified in previous conventional imaging modalities. The TNM classification system recommended by the EAU Prostate Cancer Guide was utilized for standardized evaluation of the imaging results, with particular attention paid to regional lymph nodes to differentiate between metastatic and non-metastatic disease.

### 2.3. Imaging Protocol

The imaging protocol employed in our study adhered to stringent standards to ensure accuracy and consistency. Prior to undergoing Ga68 PSMA PET-CT imaging, patients provided informed consent for the procedure. Subsequently, each patient received intravenous administration of 5.1 mCi Gallium-68 PSMA, followed by imaging conducted 60 minutes post-administration. Imaging encompassed the area from the vertex to the midfemur and was performed using Siemens Biograph PET-CT equipment. Following attenuation correction with CT, images were meticulously evaluated both visually and numerically by a single experienced nuclear medicine specialist. The maximum standardized uptake value (SUVmax) was meticulously recorded, either from visually identified lesions or from the highest area within the prostate gland.

### 2.4. Statistical Analysis

The statistical analysis employed in our study was comprehensive and rigorous, designed to extract meaningful insights from the collected data. To begin, the distribution of numerical variables was carefully assessed using the one-sample Kolmogorov–Smirnov test to determine the appropriate analytical approach. Parametric or non-parametric tests were then applied accordingly, with a predefined significance level of α = 0.05. For numerical variables, Student’s *t*-test or Mann–Whitney U test was employed, while the Pearson chi-squared test or Mann–Whitney U test was utilized for ordinal categorical variables. Furthermore, binary logistic regression analysis was conducted to identify independent risk factors for PSMA-PET positivity, followed by multivariate logistic regression analysis for statistically significant variables. The entire statistical analysis process was executed using IBM SPSS 22.0 software, ensuring robustness and reliability in our findings.

## 3. Results

Among the newly diagnosed prostate cancer patients who applied to our clinic between October 2020 and October 2021, a comprehensive assessment categorized 499 non-metastatic patients who underwent staging by 68Ga PSMA PET-CT as Group 1, while 243 patients with de novo metastatic conditions were designated as Group 2.

Comparing demographic characteristics, the mean age for Group 1 stood at 67.7 ± 7.4 years, whereas for Group 2, it was slightly higher at 70.2 ± 8.8 years, exhibiting statistical significance (*p* < 0.001). Prostate volume exhibited similar measurements across both groups. However, significant differences emerged in total PSA levels, dPSA, biopsy ISUP Grade Group (GG), cT stage determined by rectal examination, D‘Amico risk class, and SUVmax value of the prostate, all of which were notably higher in Group 2 (*p* < 0.001) (Refer to Table 1).

A breakdown of ISUP GG distribution revealed variations between the groups. In Group 1, ISUP GG1 accounted for 108 cases (21.6%), followed by ISUP GG2 with 116 cases (23.2%), ISUP GG3 with 134 cases (26.9%), ISUP GG4 with 75 cases (15%), and ISUP GG5 with 66 cases (13.2%). Conversely, in Group 2, ISUP GG1 constituted 20 cases (8.2%), ISUP GG2 had 17 cases (7%), ISUP GG3 comprised 43 cases (17.7%), ISUP GG4 had 58 cases (23.9%), and ISUP GG5 constituted the majority with 105 cases (43.2%). Notably, while biopsy ISUP GG 1, 2 and 3 were more prevalent in Group 1, higher-risk categories, GG4 and GG5, were prominently observed in Group 2 (See Table 1).

Further analysis revealed a higher prevalence of abnormal digital rectal examination findings in the metastatic group. In Group 1, the distribution of cT stages included 354 cases of cT1c (70.9%), 65 cases of cT2a (13%), 56 cases of cT2b (11.2%), and 24 cases of cT2c (4.8%). For Group 2, the distribution was as follows: 45 cases of cT1c (18.5%), 45 cases of cT2a (18.5%), 81 cases of cT2b (33.3%), and 72 cases of cT2c (29.6%) (See Table 1).

According to the D’Amico risk classification, Group 1 exhibited a higher prevalence of low and intermediate risk cases, whereas high-risk cases were more predominant in Group 2. Specifically, Group 1 comprised 53 low-risk patients (10.6%) and 222 intermediate-risk patients (45.1%), whereas Group 2 included only 5 low-risk patients (2.1%) and 23 intermediate-risk patients (9.5%). The high-risk category accounted for 221 patients (44.3%) in Group 1 and a substantial majority of 215 patients (88.5%) in Group 2 (refer to Table 1).

Multivariate logistic regression analysis, as illustrated in Table 2, identified the presence of cT2 and dPSA as independent risk factors for detecting metastasis on Ga68 PSMA PET-CT.

## 4. Discussion

Conventional imaging modalities, such as transrectal ultrasound (TRUS), computed tomography (CT), bone scintigraphy and magnetic resonance imaging (MRI), have served as the cornerstone of prostate cancer staging for decades. However, these modalities exhibit limitations in sensitivity and specificity, particularly in the detection of small metastatic lesions and lymph node involvement. In contrast, PSMA PET-CT stands out for its superior sensitivity and specificity in detecting metastatic disease, capable of identifying lesions as small as a few millimeters in size [16]. While there is ongoing debate regarding the clinical significance of detecting oligometastatic disease, which conventional imaging methods often miss, the use of PSMA PET-CT in primary staging holds immense potential to revolutionize treatment algorithms. By providing more accurate risk stratification and guiding personalized treatment strategies, PSMA PET-CT offers a promising avenue for improving patient outcomes in prostate cancer management [17]. Despite the potential benefits of PSMA PET-CT, the detection of metastases via this imaging modality may lead some patients to forego definitive treatment. However, it is crucial to recognize that treatment options for metastatic disease are continually evolving. Androgen pathway inhibitor usage is increasing rapidly, but Docetaxel is still a robust option for high-volume metastatic prostate cancer [18].

The emergence of PSMA PET-CT has significantly enhanced the diagnostic capabilities in prostate cancer staging. Offering improved sensitivity and specificity in detecting primary tumors, regional lymph node involvement, and distant metastases, PSMA PET-CT represents a significant advancement in imaging technology [19]. Turpin et al. demonstrated that PSMA PET-CT outperforms conventional imaging methods in evaluating treatment response and detecting metastases, further underscoring its clinical utility in disease management [20].

Previous studies have investigated the correlation between PSMA PET-CT findings and various clinical parameters, such as PSA levels, Gleason score, and D’Amico risk class. Uprimny et al. found a correlation between PSA levels and PSMA PET-CT uptake in a retrospective study of 90 patients undergoing primary staging [21]. In the study of Sanlı et al., in which 109 BCR cases published in 2017 were examined, there was a correlation between PSA levels and 68Ga PSMA PET-CT uptake, but no correlation was found with Gleason score [22]. In the study of Koerber et al., in which 104 newly diagnosed prostate cancer patients were evaluated, it was revealed that PSMA involvement increased as PSA, Gleason score and D’Amico risk class increased. SUVmaxes were also evaluated in this study. The average SUVmax of benign prostate areas was found to be 1.88, and the average SUVmax of malignant areas was found to be 10.77 [23]. In our study, consistent with these findings, the incidence of ISUP GG4 and ISUP GG5 in metastatic disease, PSA, and D’Amico high-risk disease were found to be statistically significantly higher. SUVmax values are also significantly higher in metastatic group.

Research consistently supports the superior diagnostic performance of PSMA PET-CT compared to conventional imaging modalities across various clinical scenarios. Studies have shown that PSMA PET-CT leads to significant changes in treatment decisions, especially in cases of biochemical recurrence where conventional imaging methods may fail to accurately detect disease spread. Additionally, PSMA PET-CT offers advantages such as reduced radiation exposure and fewer equivocal findings, leading to more precise clinical decisions [12,24,25,26]. Additionally, PSMA PET-CT offers advantages such as reduced radiation exposure and fewer equivocal findings, leading to more precise clinical decisions [12].

The ability of PSMA PET-CT to identify clinically significant prostate cancer based on SUVmax thresholds further enhances its utility in guiding treatment decisions [27]. These findings highlight the transformative impact of PSMA PET-CT on prostate cancer management and patient outcomes. Studies also showed that higher expression of PSMA itself is correlated with higher BCR [28].

Despite its promising data, the widespread adoption of PSMA PET-CT in clinical practice faces several challenges. These include the lack of standardized imaging protocols, limited availability of PSMA radiotracers, and variations in interpretation criteria across institutions [17]. Additionally, reimbursement issues and cost-effectiveness concerns may impact the accessibility of PSMA PET-CT for patients. Furthermore, the potential for overdiagnosis and overtreatment of indolent prostate cancer lesions detected by PSMA PET-CT raises clinical dilemmas that need to be addressed [29].

Our study represents a significant contribution to the field, being the only study to precisely investigate predictors of metastases in PSMA PET-CT and the largest study to investigate the relationship between clinical and biochemical features with PSMA PET-CT. A limitation of the current analysis is retrospective design. Prospective studies are needed to validate these findings and further elucidate the role of PSMA PET-CT in primary staging, risk stratification and treatment response assessment. Additionally, research exploring the role of PSMA-targeted therapies in combination with PSMA PET-CT for personalized treatment strategies is warranted. Collaborative efforts between academia, industry, and regulatory agencies are essential to address these research gaps and accelerate the translation of PSMA PET-CT into routine clinical practice.

## 5. Conclusions

PSMA PET-CT heralds a transformative era in prostate cancer imaging, boasting unparalleled sensitivity and specificity when compared to conventional imaging modalities. Its remarkable capacity to precisely identify primary tumors, regional lymph node involvement and distant metastases carries profound implications for the diagnosis, staging and treatment planning of prostate cancer. Despite encountering challenges in its widespread clinical implementation, the potential of PSMA PET-CT to revolutionize prostate cancer management and significantly enhance patient outcomes cannot be overstated.

The future of prostate cancer care hinges on concerted efforts to address these challenges, optimize imaging protocols and validate the clinical efficacy of PSMA PET-CT through large-scale prospective studies. Through collaborative endeavors involving researchers, clinicians, industry stakeholders and regulatory agencies, PSMA PET-CT holds promise to fundamentally transform the landscape of prostate cancer care, ushering in a new era of precision medicine and improved patient outcomes worldwide. With continued dedication and innovation, PSMA PET-CT has the potential to emerge as a cornerstone technology in the fight against prostate cancer, offering hope and healing to countless individuals affected by this disease.

## Figures and Tables

**Table 1:** Demographic, biochemical and clinical data of patients.

	Group 1 (n = 499)	Group 2 (n = 243)	*p*-Value
Age	67.7 ± 7.4	70.2 ± 8.8	<0.001 ^T^
Total PSA, ng/dL	10.9 (1.0–658.0)	75.3 (0.8–2305.0)	<0.001 ^M^
Prostate volume, cc	43 (9–174)	50 (20–291)	0.057 ^M^
PSA density, ng/dL/cc	0.241 (0.024–2.222)	0.666 (0.051–6.049)	<0.001 ^M^
Biopsy ISUP, n (%)			<0.001 ^M^
Grade Group 1	108 (21.6)	20 (8.2)	
Grade Group 2	116 (23.2)	17 (7)	
Grade Group 3	134 (26.9)	43 (17.7)	
Grade Group 4	75 (15)	58 (23.9)	
Grade Group 5	66 (13.2)	105 (43.2)	
cT stage, n (%)			<0.001 ^M^
cT1c	354 (70.9)	45 (18.5)	
cT2a	65 (13)	45 (18.5)	
cT2b	56 (11.2)	81 (33.3)	
cT2c	24 (4.8)	72 (29.6)	
D’Amico risk classification, n (%)			<0.001 ^P^
Low Risk	53 (10.6)	5 (2.1)	
Intermediate Risk	225 (45.1)	23 (9.5)	
High Risk	221 (44.3)	215 (88.5)	
SUVmax prostate	9.7 (2.6–98.5)	16.4 (2.9–111.6)	<0.001 ^M^

^T^: Student T test; ^M^: Mann–Whitney U test; ^P^: Pearson’s chi-squared test.

**Table 2:** Multivariate logistic regression analysis of factors affecting the detection of metastatic disease in PSMA PET-CT.

	Univariate Model					Multivariate Model				
	OR	95% CI			*p*-Value	OR	95% CI			*p*-Value
Age	1.040	1.020	-	1.061	<0.001					
Total PSA	1.018	1.014	-	1.023	<0.001					
Biopsy ISUP GG	1.910	1.675	-	2.178	<0.001					
cT stage	2.982	2.523	-	3.525	<0.001					
cT2c stage	8.333	5.085	-	13.656	<0.001	3.538	1.206	-	10.383	0.021
D’Amico risk class	6.674	4.520	-	9.853	<0.001					
D’Amico high risk	9.659	6.273	-	14.872	<0.001					
Prostat hacmi	1.009	1.000	-	1.018	0.051					
Prostate density	3.876	2.364	-	6.353	<0.001	2.990	1.781	-	5.021	<0.001
SUVmax prostate	1.043	1.030	-	1.056	<0.001					

## Data Availability

The datasets presented in this article are not readily available because the data are part of an ongoing study.
